# Progression, Symptoms and Psychosocial Concerns among Those Severely Affected by Multiple Sclerosis: A Mixed-Methods Cross-Sectional Study of Black Caribbean and White British People

**DOI:** 10.1371/journal.pone.0075431

**Published:** 2013-10-02

**Authors:** Jonathan Koffman, Wei Gao, Cassie Goddard, Rachel Burman, Diana Jackson, Pauline Shaw, Fiona Barnes, Eli Silber, Irene J. Higginson

**Affiliations:** 1 King’s College London, School of Medicine, Cicely Saunders Institute, Department of Palliative Care, Policy and Rehabilitation, London, United Kingdom; 2 King’s College Hospital NHS Foundation Trust, Neurology Department, London, United Kingdom; Kyushu University, Japan

## Abstract

**Objective:**

Multiple sclerosis is now more common among minority ethnic groups in the UK but little is known about their experiences, especially in advanced stages. We examine disease progression, symptoms and psychosocial concerns among Black Caribbean (BC) and White British (WB) people severely affected by MS.

**Design:**

Mixed methods study of 43 BC and 43 WB people with MS (PwMS) with an Expanded Disability Status Scale (EDSS) ≥6 involving data from in clinical records, face-to-face structured interviews and a nested-qualitative component. Progression Index (PI) and Multiple Sclerosis Severity Score (MSSS) were calculated. To control for selection bias, propensity scores were derived for each patient and adjusted for in the comparative statistical analysis; qualitative data were analysed using the framework approach.

**Results:**

Median EDSS for both groups was (6.5; range: 6.0–9.0). Progression Index (PI) and Multiple Sclerosis Severity Score (MSSS) based on neurological assessment of current EDSS scores identified BC PwMS were more likely to have aggressive disease (PI F = 4.04, p = 0.048, MSSS F = 10.30, p<0.001). Patients’ reports of the time required to reach levels of functional decline equivalent to different EDSS levels varied by group; EDSS 4: BC 2.7 years v/s WB 10.2 years (U = 258.50, p = 0.013), EDSS 6∶6.1 years BC v/s WB 12.7 years (U = 535.500, p = 0.011), EDSS 8: BC 8.7 years v/s WB 10.2 years. Both groups reported high symptom burden. BC PwMS were more cognitively impaired than WB PwMS (F = 9.65, p = 0.003). Thematic analysis of qualitative interviews provides correspondence with quantitative findings; more BC than WB PwMS referred to feelings of extreme frustration and unresolved loss/confusion associated with their rapidly advancing disease. The interviews also reveal the centrality, meanings and impact of common MS-related symptoms.

**Conclusions:**

Delays in diagnosis should be avoided and more frequent reviews may be justified by healthcare services. Culturally acceptable interventions to better support people who perceive MS as an assault on identity should be developed to help them achieve normalisation and enhance self-identity.

## Introduction

Geographical origin is an important modifier of multiple sclerosis (MS). MS is more prevalent among people living in the United Kingdom, Europe, and in north America compared to Asia and Africa [1] [2] [3]. Genetic predisposition, environmental factors, geographical variation in late onset Epstein Barr virus, inadequate serum concentrations of vitamin D may explain this variable prevalence [4] [5] [6]. Ethnicity is also thought to influence clinical manifestations of MS; some groups are reported to experience more aggressive disease and greater levels of disability [4] [7]. Increasing evidence indicates a growing number of people from migrant populations to the UK are developing MS [5], [8]. However, little is known about the progression, impact or psychosocial meanings attributed of their disease. Most studies to date have relied on data exclusively extracted from clinical records [4] [7]; these misdirect patient experiences of symptom impact and psychosocial concerns. Further, data is concentrated on people with MS (PwMS) in the earlier stages their disease, rather than those in more advanced stages when their needs for health and social care increases. Addressing this concern is clinically important for the following reasons. First, across the world, globalization has brought with it an unprecedented increase in the numbers of persons who have migrated to developed countries. The total number of international migrants has increased over the last 10 years from an estimated 150 million in 2000 to 214 million persons today [9]: approximately 72.1 million of these immigrants arrived in Europe and 42.8 million in North America, a tripling of the immigrant populations in these regions compared to twenty years earlier [9]. This trend is expected to continue. A greater understanding of the similarities and differences across the two ethnic groups has important implications for effective delivery of palliative care for people with progressive disease and their families. This recommendation was recently endorsed in the UK Department of Health’s End of Life Care Strategy *‘End of Life Care Strategy: Equality Impact Assessment’* [10] and in their subsequent annual reports [11], all of which highlight the importance that awareness and education in multicultural health should play a role in helping embrace society’s diversity and changing needs. Therefore, in this paper we aim to examine and explore the presence of patient-centred experiences and meanings of illness progression, symptoms, and psychological and palliative concerns among Black Caribbean (BC) and White British (WB) people severely affected by MS. We chose to compare the two ethnic groups for the following reasons. Second, just focusing on a single ethnic group can imply that they are ‘exotic’ [12] and by definition different or idiosyncratic compared to the majority population. Second, there is little published qualitative research of the accounts of WB PwMS. The comparison may therefore develop an understanding of important clinical relevant commonalities and differences between different ethnic groups.

## Methods

### Ethics Statement

Ethical and research governance approval were obtained from King’s College Hospital Research Ethics Committee (South London REC Office (2) (ref: 10/H0808/43) and three Research & Development Departments (Lambeth, Southwark, Lewisham & Bexley RDLSLBe545, Guy’s & St. Thomas RJ112/N042, and South London Healthcare SLHT/2010/UCSN/neuro/27).

### Design

We undertook a mixed methods study among White British and Black Caribbean severely affected by MS which included data collected from their clinical records based on neurological assessment, structured face to face interviews using validated measures of symptom burden, and nested qualitative interviews to explore participants’ accounts of their MS and its impact on their lives. Compared with mono-method research our rationale for this design was that the synthesis of two research methods, quantitative and qualitative, from conception, through data collection - asking distinct but intersecting questions [13] [14] [15] [16] - could be then integrated to reveal new dimensions of the multi-faceted relationship between MS and ethnicity.

### Setting

This study took place in two NHS hospital trusts and six primary care services serving a materially and socially deprived geographical area including south London and surrounding areas with a total population of 1.61 million people and with one of the highest concentrations (25%) of Black Caribbean people in the country [17]. A comprehensive MS service serves this population with a network of MS nurses based in both hospitals and the community. The majority of people with MS in this geographical area are cared for by the MS service at King’s College Hospital NHS Foundation Trust either at King’s College Hospital itself (with approximately 500 PwMS) or within services located at other district general hospitals (with approximately 1,000 PwMS). In addition there are six community MS nurses working in each of the south-east London boroughs. To minimise any possibility of not including all Black Caribbean PwMS living locally and served by other providers of care, we also recruited via a voluntary sector organisation for London’s BC community.

### Study Participants

#### Inclusion criteria

Adults aged 18+yrs as having multiple sclerosis confirmed by a neurologist based on the modified “McDonald Criteria” of the International Panel on Diagnosis of MS [18] [19], with evidence derived from clinical records based on recent neurological assessment of an Expanded Disability Status Scale (EDSS) score of ≥6.0 [20] (i.e. at least requiring a walking aid - cane, crutch, etc. - to walk about 100 m with or without resting, refer to Appendix S1), self-identified as WB or BC, with sufficient cognitive capacity to consent and complete the interviews, as determined by the referring clinicians, a score of <15 on the six-item Blessed Orientation Memory Concentration Test (BOMCT), and providing written informed consent. BOMCT scores range from 0–28, with scores of 15 and over being indicative of an increased cognitive impairment and thereby diminished capacity [13].

#### Exclusion criteria

Patients not providing consent or lacking capacity.

### Recruitment and Informed Consent Procedures

For intrinsic and situational reasons those from minority ethnic communities have traditionally been a regarded as vulnerable populations who have been exploited in medical research [21] [22]. Based on recent local research among the Black Caribbean community that examined this issue in detail [23] we designed study recruitment procedures that maximised opportunities for justice and adhered to important ethical principles that ensured a duty of care to all research participants in addition to respecting their rights and autonomy. Eligible people with MS (PwMS) from both ethnic groups were informed about the study by the neurology services during outpatient clinics, and through introductions made by MS clinical nurse specialists’ from their database of community contacts, and from a voluntary sector organisation in the region. Eligible PwMS were invited to participate in the study, were then provided with a comprehensive study participant information sheet and given 48 hours to consider their involvement. CG or JK then organised a convenient time and location (usually the patient’s home) for a face-to-face interview. Written informed consent was provided prior to beginning each interview. Participants also provided their consent to examine their clinical records. In order to eliminate any potential for coercion potential participants who declined to participate in the study were reassured verbally and in writing that their current/or future treatment and care would not be compromised in any way.

At the end of the quantitative interview all participants were asked if they would be willing to take part in a second semi-structured interview to further explore the meaning of their illness and their experiences. PwMS who agreed were purposively selected, to give a maximum variation sample by MS disease types, genders and ages and EDSS level aiming for 15 participants per ethnic group. Qualitative sampling strategies are not designed to achieve statistical generalisation or test hypotheses, instead they take an inductive to study in-depth the range and complexity of meanings and phenomena relevant to the research question, with the aim of providing explanations and conceptual generalization [24].

### Data Collection

#### The quantitative survey

After providing informed consent face-to-face interviews with PwMS were conducted by CG and JK, both trained in interviewing people with progressive disease. The questionnaire recorded standardised demographical and clinical data and information relating to participants’ functional status and symptoms. First, clinical diagnosis and patient participants’ current EDSS score based on neurological assessment were extracted from clinical records. Second, participants were asked to self-report: (i) when they first had symptoms of MS (defined as ‘onset’ of disease); (ii) when they reached different stages of function equivalent to different EDSS stages; (iii) self-assess their symptom impact, palliative and psychosocial concerns according to validated measures which were selected based on the results of a systematic literature review appraising questionnaires for use among people severely affected with MS [25]. The measures included MS specific measures (the Multiple Sclerosis Impact Scale (MSIS) [26] and the Modified Fatigue Impact Scale (MFIS) [27]) and generic measures that have shown validity among people with MS and other advanced illnesses – to assess depression and anxiety (the Hospital Anxiety and Depression Scale (HADS) [28], and palliative concerns, using the Palliative Care Outcomes Scale (POS) and POS-Symptoms (POS-S) [29] [30] (see Appendix S1 for more details).

#### The qualitative interviews

The interviews were informal in style and followed a topic guide developed but not constrained by a review of the literature [31] [32] [33]. This was further developed with help from members of a London-based MS patient advocacy service for the BC community. Questions included: “*Tell me in your own words what bothers or troubles you most about your illness”.* Prompts were used to elicit further information. All interviews were audio-recorded and lasted on average 77 minutes (range 20–120 minutes). These were recorded to expand the researchers’ impressions of the interview.

### Analysis (Quantitative)

The analysis reports and compares across both ethnic groups: (i) participants’ demographic and clinical characteristics; (ii) MS disease progression based on neurological assessment recorded in patient participants’ clinical records and their self-reports of the time taken to reach levels of physical decline equivalent to different EDSS levels; (iii) the prevalence of MS-related symptoms, severity and impact; and (iv) psychological and palliative concerns. To adjust for potential selection bias, a propensity score (PS) was calculated for each patient [34]. The PS was defined as the probability of being in the study in relation to observed differences in age structure, type of MS and EDSS scores between the two groups of participants. The PS score was derived by using logistic regression using the following variables: ethnicity, age, type of MS and EDSS score.

We determined MS progression rates by: (i) calculating the progression index (PI) (current EDSS score as recorded in participants’ clinical records divided by years since their clinical diagnosis) [35] and (ii) participants’ reports of when they reached different stages of functional decline equivalent to different EDSS stages. However, there is no fixed consensus on the methods for measuring MS disease progression using single, cross-sectional assessments of disability [36]. Another approach includes the Multiple Sclerosis Severity Score (MSSS) [37] which also incorporates the EDDS score and the duration of illness from the point of clinical diagnosis, but then compares an individual’s disability with the distribution of scores in cases having equivalent disease duration. We conducted sensitivity analysis to addresses the question whether our findings are robust when compared to other methods.

Differences in PIs, MSSS, severity of fatigue, pain and depression by ethnic group were analyzed using analysis of covariance (ANCOVA), with PS being included as a covariate. We also estimated the correlation coefficient between the PI from diagnosis and the score of BOMCT. Statistical significance (two-sided) was set at p<0.05.

### Sample Size

There were no other available UK data on the distributions of disease progression or symptoms among BC PwMS on which to base this study. A UK clinical study suggested unusually rapid progression among UK-born West Indians [39] supporting local experience, but did not quantify the effects. Therefore prior to data collection commencing we used a moderate effect size of 0.65 to estimate the sample size required to detect a difference in MS progression between BC and WB PwMS. With a power of 80% and an alpha level of 0.05, for a two-sided, two-sample t-test, we estimated we would need to recruit at least 39 participants in each group to detect a difference. To identify potential differences in MS symptom distress we made use of distributions from a recent comparative survey of BC and WB patients with advanced cancer where severe cancer pain distress varied between 89% for BC and 55% for WB patients [40]. With power set at 80% and an alpha level of 0.05 a minimum of 32 cases would be required from each group to detect a similar difference. As the study was primarily exploratory our sample size of 43 patients per group was able to explore these clinically important differences, and allow for a small amount of missing data.

### Analysis (Qualitative)

All qualitative interviews were transcribed verbatim, anonymised, and analysed using the framework approach [38] with the aid of QSR Nvivo (Version 8.0) to explore in detail participants ‘accounts of their illness progression and MS-related symptoms. This matrix-based approach involved developing a thematic framework through the independent repeated and detailed readings of the raw data. The interview segments in which participants discussed their MS illness progression and symptoms were then summarised. Broad themes were identified and further refined before being entered into a matrix. This facilitated exploration of relationship between themes within and across cases. To address issues of analytical rigor and trustworthiness the data from interview transcripts were examined by CG and then discussed in detail with JK. Coding discrepancies were discussed by the two researchers until consensus was reached. We also paid attention to deviant or non-confirmatory cases where emerging themes contradicted more common ideas. Participants’ ethnic identities are preceded by ‘BC’ (for Black Caribbean) and ‘WB’ (for White British).

## Results

We approached 97 PwMS, 46 BC and 51 WB PwMS; 43 (93%) BC and 44 (86%) WB PwMS agreed to participate. One WB PwMS was subsequently excluded from the study after scoring over 15 with the BOMCT, leaving us with a total of 86 (43 BC, 43 WB) participants. Reasons for declining participation included those whose illness progressed and became too unwell to interview, complex family circumstances, and those who did not express an interest in the survey. Most participants were born in the UK, apart from 9 in the BC group (table 1). Over two-thirds were women (60/86). BC participants were younger (mean ages, BC 47.7 yrs, WB 57.5 yrs, t-test = 3.97, p<0.000). MS disease types were: relapsing-remitting 28% (n = 24); secondary progressive 37% (n = 32); primary progressive or progressive relapsing 35% (n = 30). Twice (16 vs 8) as many BC as WB PwMS were diagnosed with relapsing-remitting MS. WB PwMS were diagnosed with MS for longer periods than their BC counterparts (Mean 16.1 yrs; vs 10.2 yrs; t-test = 3.01, p = 0.003). Four WB were treated with disease modifying therapy (DMT) compared to 12 BC PwMS. Medication included glatiramer acetate (n = 3), natalizumab (n = 3), interferon beta therapy (n = 9) and unspecified (n = 1). Two PwMS, one per ethnic group, were recorded as receiving chemotherapy (table 1).

**Table 1:** Demographic and clinical characteristics of Black Caribbean and White British participants included in the study.

	White British (n = 43)	Black Caribbean (n = 43)	P value for statistical test
**Place of birth:**			
United Kingdom	43 (100%)	34 (79%)	Fisher’s exact p = 0.003
Caribbean	0	9 (21%)	
**Gender:**			
Male	15 (35%)	11 (26%)	Chi-sq 0.88 (df 1), p = 0.35
Female	28 (65%)	32 (74%)	
**Mean age, SD (range) Median**	57.5 yrs, 12.1 (35–88) 57.7	47.7 yrs, 10.8 (27–75) 48.1	t-test = 3.98(df 84), p<0.001
**Highest level of education attained:**			
Did not go to school	1 (2%)	0	Fisher’s exact p = 0.053
Primary school	1 (2%)	0	
Secondary School (GCSE)	13 (30%)	20 (47%)	
Secondary School (A-level)	8 (19%)	10 (23%)	
University	20 (47%)	9 (21%)	
**Degree/equivalent professional qualification:**			
Yes	23 (53%)	9 (21%)	Chi-sq 9.75 (df = 1), p = 0.002
No	20 (47%)	34 (79%)	
**Geographical area of residence - Indices** **of multiple deprivation (IMD) (grouped):**			
Group 1 (most deprived)	22 (51%)	23 (56%)	Fisher’s exact p = 0.46
Group 2	16 (37%)	18 (42%)	
Group 3	3 (7%)	0	
Group 4 (least deprived)	2 (5%)	2 (5%)	
**Religion:**			
No religion	12 (28%)	5 (12%)	Fisher’s exact p = 0.040
Christian	26 (60%)	33 (76%)	
Buddhist	5 (12%)	2 (5%)	
Other	0	3 (7%)	
**Type of MS:**			
Relapsing remitting	8 (19%)	16 (37%)	Fisher’s exact p = 0.14
Secondary progressive	19 (44%)	14 (33%)	
Primary progressive	16 (37%)	14 (33%)	
**Age at:**			
Onset mean yrs, SD	34.3 yrs, 13.9	34.1 yrs, 11.2	t-test 0.08, p = 0.94
Diagnosis mean yrs, SD	41.4 yrs, 14.3	37.4 yrs, 11.3	t-test 1.43, p = 0.16
**MS duration from diagnosis:**			
Mean yrs, SD (range)	16.1 yrs, 11.2 (1.67–0.29)	10.2 yrs, 5.7 (0.89–23.27)	t-test 3.01, p = 0.004
Median	12.4	9.5	
**EDSS score:**			
6.0	15 (35%)	10 (23%)	Fisher’s exact, p = 0.77
6.5	9 (21%)	14 (33%)	
7.0	4 (9%)	6 (14%)	
7.5	3 (7%)	4 (9%)	
8.0	8 (18%)	6 (14%)	
8.5	2 (5%)	1 (2%)	
9.0	2 (5%)	2 (5%)	
**Time taken to reach EDSS stage from onset** **of symptoms (self- reported):**			
EDSS 4.0 mean yrs, SD (range) median	13.1 yrs, 12.0 (0.0–48.2) 10.2	5.9 yrs, 6.4 (0.0–25.6) 2.7	U = 258.5, p = 0.013
EDSS 6.0 mean yrs, SD (range) median	14.4 yrs, 11.9 (0.6–51.6) 12.7	7.6 yrs, 6.3 (0.9–25.9) 6.1	U = 535.5, p = 0.011
EDSS 8.0 mean yrs, SD (range) median	15.3 yrs, 13.9 (2.0–43.0) 10.2	12.0 yrs, 6.7 (5.9–24.6) 8.7	U = 95.0, p = 0.5

For the qualitative component we interviewed 30 PwMS; 5 men and 10 women from each ethnic group. Similar to the main survey, BC participants were typically younger than their WB peers and had been living for a shorter period of time with their MS (median 9 years v/s median 18 years). While most BC participants were second generation, two participants had moved to the UK from Jamaica aged twelve and nine years.

### MS Disease Progression and Meaning Making

The results from calculating the Progression Index (PI) and Multiple Sclerosis Severity Score (MSSS) based on patients participants’ current EDSS score extracted from their clinical records are presented in table 2 and identify similar findings. Both reveal statistically significant differences across the two ethnic groups after compensating for the propensity score, which adjusted for participants’ ethnicity, age, type of MS and EDSS at time of interview (PI test result F = 4.04, p = 0.04; MSSS test result F = 10.30, p<0.001). Participants’ reports of the time required to reach different stages of functional decline equivalent to different EDSS levels also differed by ethnic group (table 1). The median time reported to reach EDSS 4 (being able to walk without aid or rest for up to 500 metres) was 2.7 years for BC PwMS compared to 10.2 years among the WB patient group (Mann Whitney U = 258.5, p = 0.013). Thereafter the gap narrowed. The median time until needing unilateral assistance for ambulation (EDSS 6) was 6.1 years among BC PwMS compared to 12.7 years among their WB peers (Mann Whitney U = 535.5, p = 0.011). BC PwMS reached EDSS 8 (restricted to bed, chair or perambulated in a wheelchair) stage after a median time of 8.7 years and the median time WB PwMS was 10.2 years (Mann Whitney U = 95.0, p = 0.5).

**Table 2:** Comparison of disease progression and impact of symptoms among Black Caribbean and White British participants severely affected by MS.

Measure	N		Score		ANCOVA results*	
	BC (n = 43)	WB (n = 43)	BC (n = 43)	WB (n = 43)	F value	P value
**Progression Index (current EDSS based** **on clinical assessment/time clinical** **diagnosis in yrs):**						
Mean, SD (range) median	42	43	0.74, 0.9 (0.16–5.50) 0.55	0.46, 0.4 (0.08–1.85) 0.39	4.04	0.048
**Multiple Sclerosis Severity Score** **(MSSS) (current EDSS based on clinical** **assessment/time clinical diagnosis in yrs):**						
Mean, SD (range) median	42	43	8.80,1.11 (5.35–9.93) 9.32	8.23, 1.37 (5.16–9.94) 8.47	10.30, 1 df	0<0.001
**POS (i):**						
Total score, SD (range)	43	42	12.79, 6.6 (1–28)	11.09, 5.8 (1–30)	1.93	0.17
**POS-S (i)**:						
Total score, SD (range)	42	43	17.48, 10.1 (3–42)	17.95, 8.9 (3–44)	0.03	0.86
**MSIS-29 (ii):**						
Psychological subscale, SD (range: 0–100)	43	43	36.24, 23.6 (0–88.9)	33.98, 19.8 (2.8–94.0)	0.42	0.52
Physical subscale, SD (range: 0–100)	42	43	54.46, 18.9 (10.0–95.0)	54.16, 19.6 (21.3–93.8)	0.03	0.85
Total score, SD (range)	42	43	85.74, 20.7 (38.0–118.0)	84.56, 21.2 (50.0–138.0)	0.16	0.69
**MFIS (iii):**						
Physical subscale, SD (range 0–36)	43	43	19.51, 8.1 (3–36)	22.33, 6.9 (3–35)	2.78	0.10
Cognitive subscale, SD (range 0–40)	43	43	15.30, 10.1 (0–37)	14.88, 8.5 (0–40)	0.11	0.74
Psychosocial subscale, SD (range 0–8)	43	43	4.04, 2.2 (0–8)	4.44, 2.5 (0–8)	0.43	0.51
Total score, SD (range 0–84)	43	43	38.86, 19.0 (4–77)	41.65, 15.6 (5–83)	1.98	0.14
**BOMCT (iv):**						
Total score, SD (range 0–28)	43	43	5.91, 4.1 (0–14)	3.41, 3.8 (0–14)	9.65	0.003
**HADS (v):**						
Anxiety subscale, SD (range: 0–21)	43	43	5.79, 4.2 (0–16)	5.35, 4.1 (1–17)	0.42	0.52
Depression subscale, SD (range: 0–21)	43	43	6.40, 4.2 (0–17)	5.58, 3.7 (1–14)	1.23	0.27
Total score, SD (range)	43	43	12.19, 7.8 (0–30)	10.93, 6.8 (2–31)	0.94	0.33

(i) Palliative Care Outcome Scale (POS) & POS-S symptoms: comprises 10 items on anxiety, PwMS and informal caregiver concerns, practical needs and 18 questions specifically relating to MS symptoms.

(ii) Multiple Sclerosis Impact Scale (MSIS): 29 questions on a variety of MS-related impacts of the illness.

(iii) Modified Fatigue Impact Scale (MFIS): Examines perceptions of functional limitations that fatigue.

(iv) Blessed Orientation Memory Concentration Test (BOMCT): Examines presence of cognitive impairment and thereby diminished capacity.

(v) Hospital Anxiety and Depression Scale (HADS): Assesses psychological distress, with two sub-scales of anxiety and depression, used widely among people with physical illnesses.

* The analysis of covariance (ANCOVA) was adjusted for the probability of participating in the study (propensity score).

When asked, in the qualitative interviews, what ‘bothered’ or ‘troubled’ participants most about their illness, more (n = 8) BC PwMS than WB PwMS (n = 3) volunteered views on the rapidity of their illness progression and dramatic decline. This was underscored with feelings of disbelief, frustration, loss of control, and a pervading sense of grief from their sudden loss of physical function that was often extremely difficult to accommodate. When probed in more detail on the relentless nature of his ‘attacks’ a 40-year-old Black Caribbean man with relapsing remitting MS initially took his time to reflect on the pace and consequences of having to make sense of his albeit temporary periods of equilibrium which formed a brief chapter during his illness. He stated:

*Oh, (participant pauses then sighs) every couple of weeks at the moment. So it’s very frequent. Feels, feels horrible, feels **really** horrible. It brings me down. It makes my depression worse, but like that’s something else which I’ve just got to accept.* (BC PwMS 36)

Frustration and fear were common emotions BC PwMS voiced in relation to their ‘fast’ or ‘quick’ disease. This emotional state was sometimes amplified by family or friends’ perceptions of their decline; their observations acted to validate what many PwMS already knew all too well, that they were deteriorating quickly. A 48-year-old black Caribbean woman with primary progressive MS, illustrates this sentiment, but en route suggested that without her faith her situation would have been untenable:

*It’s just a lot of frustration. you know. It’s just a lot of frustration and it, it, it does play with your mind as well. It had a lot of psychological effect. because I wasn’t **always** in a wheelchair and. you know, people have commented on, you’re, you’re getting worse. You were on sticks to crutches, now to the wheelchair. And its playing on your mind and you’re thinking “Oh OK, maybe I am getting worse”. I just thought, I’d have gone right down. It was, it was that quick. …It was very scary. …**Even **in this situation my faith has played a big part, otherwise I think I would I probably would have deteriorated a **lot** more to what I, I have been.* (BC PwMS 79)

There were reports of multiple losses. Some Black Caribbean participants spoke of increasingly being denied possibilities of engaging in previously taken-for-granted activities, some of which were consider essential to help cement together human relationships. The was vocalized by a 27-year-old Black Caribbean woman with relapsing remitting MS, when she reported that her illness had robbed her of the opportunity to form an incalculable bond with her daughter during breast-feeding. She stated:

*I got sick **so** quickly. I lost the feeling down all the left side of my body …and then I was OK. And then after about a year… a month and a half after my daughter being born I got sick again. But I got worse. I had to go back on, on the treatment. I couldn’t stay off the treatment. I wanted to breastfeed her (daughter) for as long as I could, but I couldn’t, I couldn’t do it. You’re. you’re upset and you’re, you’re hurting but you don’t want to explain it to other people or see other people, see that you’re hurting, you’re crying or, I, I just, I didn’t want that.* (BC PwMS 65)

Participants’ biographies were frequently disrupted by their fast and at times steep descent. The need to take stock of events was illustrated by a 60-year-old year old Black Caribbean woman with secondary progressive MS. In her account she mentioned that the space and time required to make sense of change, a common task associated with grief work, was viewed by her clinical nurse specialist more as a routine chore, rather than recognizing the non-computable reality of the self she was obligated to leave behind as being very painful. She said:

*Yeah, every now and again I have to let myself really grieve. You’re having to go through that bereavement process again and again and quicker and quicker. Every time you lose another bit of you, you have go through it again. And I remember. the MS nurse saying to me. “I would have thought you’d have got used to this by now”. But it is **bereavement**. When I was training counsellors, we call it ‘ambiguity death’, where the person’s still here but because of what’s happened to them, the old them isn’t here anymore.* (BC PwMS18)

Rapidly changing circumstance and associated loss were also voiced by some White British participants who similarly alluded to speed of their decline. However, the desire to grieve and make sense of events was sometimes as frustrating as the catalysts that preceded these essential responses. In the following example the episodes or installments were so tightly packed that any period of acclimatization through periods of stability were challenged. A 45-year-old White British woman with primary progressive MS said:

*And the Dr said to me “You know it’s aggressive. It does get aggressive when it wants to.” “Primary’s bad enough but when it gets aggressive, it’s very aggressive.” Everything, everything happened really fast and I didn’t have a chance to get used to one thing going wrong before the next thing came along, and I, I’ve, I never had a chance to. mourn for the life I’d lost. I never had that chance cos everything was happening to quickly*. (WB PwMS 8)

### MS-related Physical and Psychological Symptoms

Table 2 shows the profile of the symptoms associated with MS across both ethnic groups as measured by three tools. Using the POS-S we identified that BC and WB PwMS both reported a mean of nine symptoms (BC PwMS mean 8.6; SD: 3.4; range 2–15 v/s WB PwMS mean 9.1; SD: 3.3; range: 2–16. Fourteen out of the 18 symptoms were reported by participants in both groups as being ‘severe’ or ‘overwhelming’. This was especially so for fatigue, pain and problems with ambulation. After adjusting for age, type of MS and EDSS, the reported severity of all symptoms were similar, the exception being pressure sores, being more of a concern among BC PwMS (F = 5.68; p = 0.019).

There were no differences between the two groups using the Multiple Sclerosis Impact Scale-29 (MSIS-29); BC PwMS reported a mean of 22 areas associated with MS impact (range 7–29) compared to 23 areas among WB PwMS (range 10–29). Fatigue, as measured by the MFIS, and depression and anxiety, as measured by HADS, were also similar between groups (table 2). Conversely, cognitive impairment differed between groups – with Blessed Orientation Memory Concentration Test (BOMCT) scores for BC PwMS more than 1.5 times greater than their WB counterparts (F = 9.65, p = 0.003, table 2). There was a weak correlation between the PI from disease onset and the score of the BOMCT (Pearson r = 0.18, p = 0.10; Spearman ρ = 0.23, p = 0.039).

Analysis of qualitative interviews identified that depression and anxiety, pain, fatigue and memory loss/confusion were common issues that caused considerable distress for participants from both ethnic groups (table 3). As with the quantitative survey findings participants reported symptoms that co-existed. Below we present findings that explore the meanings and impact of three symptoms; (i) fatigue, a symptom that was reported as being more distressing among White British than Black Caribbean participants as recorded in quantitative survey; (ii) depression, although not highlighted specifically by the HADS as being a clinically significant issue, it was nevertheless one of the most frequently mentioned concerns during qualitative interviews; and (iii) memory loss/confusion, a symptom identified BOMCT as being an issue more specific to Black Caribbean participants. These issues are presented under the following themes; ‘Unplugged, hollow and empty’, ‘Scared of living’, and ‘Everything’s like a sieve.’

**Table 3:** Concerns reported by Black Caribbean and White British participants severely affected by MS.

MS related symptoms*	Black Caribbean (n = 15)	White British (n = 15)
Depression and anxiety	6	3
Pain	5	4
Fatigue	4	4
Urinary incontinence	4	4
Stiffness	4	2
Spasms and tremors	4	1
Cognition, memory and confusion	3	1
Altered balance	2	1
Sores	1	0

* Participants reported more than one symptom.

### Unplugged, Hollow and Empty

One of the main symptoms related to severe MS reported by all participants was fatigue. They described the physical sensations of fatigue in terms of feeling ‘tired’, ‘sleepy’, ‘dozy’ ‘empty’, ‘weak’, ‘lacking in energy’ and ‘lethargy’. Beyond these words, metaphorical descriptions were used to describe the physical and mental sensations of their fatigue experience. These metaphors corresponded with lived and occasionally more abstract entities, for example, a 69-year-old White British woman with secondary progressive MS, referred to her lack of energy as *‘running out of steam’*, whilst a 60-year-old year old Black Caribbean woman, also with secondary progressive MS, described the phenomena as being akin to the *‘pulling out of electrical plugs’*. She commented:

*Although other symptoms are more frightening if I didn’t have the fatigue I’d be okay…But it’s not getting tired slowly, it’s like somebody pulled out a plug… someone pulled the plug and, phew, it’s gone. So I say living with MS is like living on a mobile phone battery. Every task I do uses up the battery. When it (the MS) hits its like somebody pulled the plug. Nothing’s happening at all, forget it, you know! And you could end up with the house in an absolute mess, your room in an absolute mess, your papers in an absolute mess, not being able to find anything that you’ve got to pay the bills you’ve got to.* (BC PwMS 18)

Some participants experienced considerable difficulty identifying and then describing their experiences. A 40-year-old Black Caribbean man with relapsing remitting MS, hesitated for a moment before attempting to convey his experience. He then managed to provide a powerful evocation to help explain that his fatigue went well beyond merely feeling tired:

*…It feels like you’ve got weights all over you. You know, it’s like you get up of a morning and it can feel like you’re just dragging lead weights around with your arms and your legs and like attached to your body which is pulling you down. You get to a chair and you flop basically, and **everything’s** like a mission. Oh I’ll make myself a cup of tea, I’ll do it in a minute. I’ll turn the TV on, I’ll do it, so you really have to force yourself to get up to turn on, to make a cup of tea, turn on the TV. Sometimes can’t be arsed to make any breakfast. You know, when you wake, do, wake up, you feel totally like hollow, just feel empty. A lot of times, I just go back to bed, to be honest.* (BC PwMS 36)

### Scared of Living

Participants reported psychologically based symptoms including depression, feeling low and anxiety. Six of the 15 BC and three of the WB participants reported their moods were low or were depressed. A smaller number from both ethnic groups reported anxiety-based symptoms. Participants made use of a variety of terms to describe these emotional states. These included feeling: ‘depressed’, ‘low’, ‘sad’, ‘grey’, ‘down’, ‘lacking motivation’’ irritable’ and ‘being down in the dumps’. A 46-year-old BC woman with secondary progressive MS provided a melancholy evocation to comprehend her periods of low mood, vacillating between recollections of being vital, and her current position, imprisoned in her chair. She said:

.. *I don’t go through depression like the MS nurse was asking me, but I **do** get depressed. There are days when I feel sad that I have MS, because I feel like I could do better. I mean I’ll even give you a confession. I was watching a Gene Kelly film the other day, and I love Gene Kelly, anyone who can dance. But at the same time I like, I hate them because I would love to do what they do. But I can’t. All I can do is just watch. I sit in the chair. I can’t actually physically get up and actually dance, because I know I’m going to flat on my face and my son will probably say “What was that for mum? And you know you can’t do that.”* (BC PwMS 15)

A 43-year-old White British man with secondary progressive MS at first denied he was in any way ‘depressed’ according to any formalised diagnostic criteria because he was concerned about the stigma associated with this label. As his account progressed however, he alluded to the bleakness of his illness, the loneliness is had bestowed on him, and the future he was required to inhabit for perhaps another two decades. His reference to *‘licking the light sockets’* may represent a means of escape from his intractable situation:

*Feeling depressed… Um. it is a. a phrase that I often think about but I don’t say, say to anyone because they’ll just think: “oh depression”, or whatever. I haven’t actually said it, but there are times I think I’m not scared of dying, I’m scared of **living**. I’m thinking if I’m like this for another thirty, I’ve been like this for twenty years, if I’m like this for another thirty, God, I’ll be licking the light sockets…* (WB PwMS 37)

### Everything’s Like a Sieve

Although a relatively under-reported symptom, cognitive problems, that included confusion and memory loss, were mentioned more often by Black Caribbean than White British participants during the qualitative interviews, supporting the quantitative findings. The principal emerging issues with memory loss and confusion were that it impacted not just on participants, but also had consequences for others; their worry was for the inconvenience it created for their family, friends or for colleagues for those who worked. Both the 40-year-old Black Caribbean man with relapsing remitting MS and a 49-year-old White British woman with relapsing remitting MS, were at pains to point out that they believed the fall-out of frequently forgetting critical events was irritating and distressing: They said:

*I always used to have a very good memory. Now my memory’s very, very poor, very poor. Long term memory’s fine but. my short term memory is really, really bad, and that’s really upsetting. Cos like I could have a talk with a friend today and arrange something for tomorrow and if I haven’t written it down, it will go straight out of my head. I won’t know until someone phones up, not happy, cos they’d have been standing somewhere for ages waiting for me. So that’s very upsetting.* (BC PwMS 36)

*The memory thing, I can’t get used to it. Because I have to remember so much for my work … what the students are doing and, and … But everything’s like a sieve. But I’ve just had to say to them “Look I. really at the moment I really can’t remember things and I am forgetful”.* **(**WB PwMS 32)

## Discussion

This mixed-method study has three main findings. First, disease progression appears to be quicker among BC than WB PwMS, but this gap narrows in more advanced stages. This result was consistent when measured in different ways, and after adjusting for demographic and clinical differences across the two ethnic groups. There is no consensus method for measuring progression in MS using single, cross-sectional assessments of disability [37] [36]; other approaches are available that incorporate similar variables [37]. We therefore adopted a well-established approach, the Progression Index (PI) [35], that has previously been used when comparing different ethnic groups [7] [41]. Moreover, we compare the results of the PI and compared this with the results of our sensitivity analysis using the Multiple Sclerosis Severity Score (MSSS). Both reveal similar findings. We calculated the PI and the MSSS based on neurological assessment of participants’ current EDSS scores and using a reliable time point, the date of their clinical diagnosis. However, since the PI may be misleading when used for very short or very long disease durations [7] we also examined the time taken from onset for all participants’ reports of the time take to arrive at level of functional decline equivalent to EDSS levels 4, 6 and 8. BC PwMS arrived at these levels significantly sooner than their WB counterparts. This more severe course is found in African-Americans as opposed to Caucasian-Americans [4] [42] [43] [44]. Possible reasons include later perceptions of symptom onset, lower socioeconomic status, genetic predisposition to more severe disease [45], and inadequate exposure to vitamin D [6]. Recent MRI comparisons suggest this may be due to higher lesion accumulation, rather than pronounced brain volume decrease [46]. However, we also found that the progression gap appears to narrow, and was less pronounced in more advanced stages (only 1.5 years to EDSS 8, compared with 7 years to EDSS 4). Reasons for this later reduction in difference between BC and WB groups warrants further investigation. Possible explanations include that: MS follows a different trajectory in BC patients, with rapid progression in early disease, which slows later, while the WB group progress more quickly; or that some BC PwMS had died before or at the time of reaching EDSS 8, confounding the result; or the that the delayed diagnosis/less use of disease modifying therapies led to a more rapid progression affecting earlier stages, but not later stages. Further, our sample for this analysis was smaller and so may be less reliable. Nevertheless, our findings point to the need for a better understanding of the trajectories of MS in the newly emerging African and Caribbean populations with longitudinal study.

The qualitative interviews correspond with the quantitative findings; we found considerable distress from loss associated with rapid decline in physical and mental function. Although loss has been reported as a common concern among PwMS [47] which continues into advanced disease, our study suggests that these losses are especially pronounced among BC PwMS who experience the more rapid decline. Clinicians need to take account of the fact that PwMS may be experiencing continuing losses and changes to identity. The study suggests that some individuals experienced disruption to their lives as a consequence of their aggressive disease, whereas others also experienced MS as an assault to their identity. MS engendered physical, emotional and social changes to the extent that some participants became a stranger unto themselves through continued losses of self. This finding supports Charmaz’s suggestion that *‘loss of self’* is experienced by people with chronic and progressive illness, for example MS, because their former actions, lives and selves are now radically precluded by illness, and where intimations of death become more pronounced [48]. The study therefore has relevance for care and support for those who experience MS as a threat to identity. Health care professionals should consider supporting these people to retain relevant aspects of pre-diagnosis identities, and also to develop new meaningful post-diagnosis identities in the context of their rapidly changing circumstances [49]. Interventions to support this approach include cognitive behavioural therapy (CBT) which appears to be successful in the early stages of MS [50], but has not been tested in more advanced illness when losses become multiple, or across ethnically diverse populations. Further, palliative care clinicians are trained in dealing with the multiple losses of progressive illness, as well as symptom control. However, they rarely care for people with MS, although a recent randomised trial, found that short-term palliative care reduced symptoms, caregiver burden and reduced health care costs [51].

Second, BC PwMS were more likely to experience higher levels of cognitive impairment than WB PwMS. The meanings and impact of this concern triangulate with the qualitative accounts provided by a small number of Black Caribbean participants and also spoke of other psychological issues including depression. Concerns regarding cognitive issues among African Americans severely affected by MS have previously been reported [52]. More recent research conducted among African American children and young adults with paediatric-onset MS have been identified as being at higher risk of cognitive impact [53]. It has been hypothesised that increased physical disability at an earlier age from more rapid disease progression may lead to greater cognitive impairments in affected individuals [52]. What remains unknown at this point, however, is to what extent this observed difference continues over time across different ethnic groups. Consequently, the observed differences may not be maintained over time, or may be maintained but at a less significant level, or may increase.

Third, we found high symptom prevalence and burden across both ethnic groups with participants experiencing an average of nine symptoms according to the POS-S [29] [30]. Most symptoms reported were considered by participants’ self-reports to be ‘severe’ or ‘overwhelming’. This was particularly so for fatigue, pain, and problems with ambulation, a finding supported by recent UK research examining symptom prevalence and severity among people severely affected by MS [54]. Whilst fatigue featured high as a concern during the qualitative interviews, psychological distress was also evident. Depression is a very common psychological problem associated with MS [54]. We observed that manifestations of distress associated with depression were more frequently voiced among BC than WB PwMS. However, we are mindful that its cause is complex and multi-factorial; depression may be induced by biological or psychosocial reasons, or through medication, including interferon (IFN) [55] [56], a disease modifying treatment that was prescribed in more instances among Black Caribbean participants. Overall, we observed few symptom related differences across the two ethnic groups, apart from pressure sores. Research evidence from a similar, although much smaller USA-based study supports our finding [57], or has identified significant racial/ethnic differences across groups with Latino and African Americans reporting less severe MS symptoms than White American PwMS. However, the range of symptoms examined in these studies were limited and relied exclusively on registry-based data [58] which is likely to result in under-reporting. BC severely affected by MS complained more about the presence of pressure sores than WB PwMS; research from the USA found that African American residents with MS admitted to care homes were almost twice as likely than white residents to have stage 4 pressure ulcers at admission that caused considerable distress [52]. This difference may be related to the earlier onset of ambulatory problems among the BC groups.

### Study Limitations

There are several limitations to our study. First, ‘racialised differences’ between research participant and interviewer may potentially affect the veracity of participants’ illness accounts [59]. Matching interviewer and participant on characteristics such as ethnicity (CG and JK are both White British), gender, age, and social class may build rapport [60]. Despite this we were successful in recruiting and interviewing PwMS, in the more advanced stages of diseases, across both ethnic groups. While most MS studies on ethnicity rely on clinical record data records [4] [7], we collected patient reported assessments from 43 BC and 43 WB patients, with further qualitative interviews amongst 30 of them. Nevertheless, we cannot exclude the possibility that more detailed qualitative data may have been found from the BC group using different researchers.

Second, the PI, an indicator of disease progression from the onset of clinical symptoms to the time of the EDSS assessment, has several limitations: (i) it can be skewed toward higher scores by short disease duration and toward lower scores by long disease durations, and (ii) the EDSS is an ordinal measure and differences between each level are not comparable [36]. That is, moving from EDSS 1.0 to 2.0 is very different to the transition from 6.0 to 7.0.

Third, we acknowledge patient participants’ recollections of the different time points associated with functional decline comparable to different EDDS levels may be subject to recall bias. In our study we believe this limitation applies to both ethnic groups. Alternative strategies where there has been a suggestion of ethnic differences in recall in the onset of MS symptoms have included asking white patients to specify the month of initial symptoms and African American patients the season when onset occurred [61].

Fourth, the health-related measures may not have cross cultural equivalence [62]. This would assume a common understanding between BC and WB PwMS in relation to the meanings, presentations and expressions underlying the physical and psychological symptoms associated with their disease; recent qualitative research has identified differences in the culturally-laden expression of pain and other symptoms among BC and WB people living with advanced cancer [32]. The charges of ethnocentricity can be reduced by examining an instrument’s performance and acceptability within a new population. Although many of the tools we used have been heavily used among other cultural groups, none have specifically been explored within BC PwMS. Complementary approaches might therefore be required to explore the experience and expression of MS symptoms that may potentially be missed using standardised tools.

Fifth, the main focus of our study was to examine the patient-centred experiences in the more advanced stages of MS (EDSS ≥6) and their progression. In such instances a cross-sectional design was appropriate to maximise recruitment and then involve participants in sharing their accounts. We acknowledge that a longitudinal study incorporating survival analysis would provide important answers to changes in physical and psychological symptoms over time and differences in clinical outcomes, including disease progression, across ethnic groups. However, this approach represents a challenge; there is currently very little clinical research among people from different ethnic, principally because of difficulties in recruitment [63]. This is attributed to cultural mistrust, paternalism where minority groups are considered to be intrinsically or extrinsically vulnerable, the perceived additional costs of recruitment, and racially-based stereotypes and myths about attitudes towards research by minority groups [23] [63]. Deliberate or inadvertent exclusion raises issues of justice. Most previous MS studies on ethnicity have therefore relied on clinical records [4] [7] which do not adequately capture patients’ report of symptoms or psychosocial concerns.

## Conclusions

This first comparative mixed-method study examined Black Caribbean and White British people severely affected by MS in terms of disease progression, symptoms, and the patient-centred meanings associated with these issues. Our findings demonstrated more aggressive MS disease among Black Caribbeans despite a number of them being in receipt of disease modifying therapy. More rapid disease progression leads to marked difficulties with ambulation, physical function and distress as a result of multiple losses. This study has implications for MS and palliative care services, in particular the development of appropriate interventions to support those people who experience MS as an assault on their identity to achieve normalization, and to maintain relevant and patient-centred pre-diagnosis ‘sense-of-self’. However, to achieve this services must be mindful to invest appropriately in training to enhance knowledge and skills in ‘cultural relativism’ or ‘multiculturalism’ [64]. These two concepts can be understood to be *“a social-intellectual movement that promote the value of diversity as a core principle and insists that all cultural groups be treated with respect as equals”* [65]. As the number of people from BAME communities affected by MS continue to increase prospective research studies must continue to examine disease progression and symptoms and their impact in more detail. Further new ways of managing the multiple losses, distresses and symptoms need to be explored, with new joint-services between neurology and palliative care and/or cognitive therapy.

## Supporting Information

Appendix S1 **Measurement instruments used in the study.**
(DOCX)
