# Supplementary material for: Progression, Symptoms and Psychosocial Concerns among Those Severely Affected by Multiple Sclerosis: A Mixed-Methods Cross-Sectional Study of Black Caribbean and White British People
Source: PLoS One. 2013 Oct 2;8(10):e75431. doi: 10.1371/journal.pone.0075431 (PMC3788806; doi:10.1371/journal.pone.0075431)
Supplement: Appendix S1 — Measurement instruments used in the study. (DOCX) [file pone.0075431.s001.docx]

**Supporting appendix S1: Measurement instruments used in the study**

| **Measure** | **Focus** | **Interpretation** |
| --- | --- | --- |
| Expanded disability status scale (EDSS) [20] | 10-point scale based on clinical examination and functional assessment to identify the level of MS disability. | Higher scores indicative of greater levels of MS-disability. A score of 6 (cut off for inclusion in this study) means “Requires a walking aid - cane, crutch, etc - to walk about 100m with or without resting” and a score of 7 means “Unable to walk beyond approximately 5m even with aid. Essentially restricted to wheelchair; though wheels self in standard wheelchair and transfers alone. Up and about in wheelchair some 12 hours a day” |
| Multiple Sclerosis Impact Scale (MSIS) [26] | Measures physical and psychological impact of MS from the patients' perspective; it comprises questions that examine the presence and impact of 20 physical issues and 9 psychological issues. | Total score ranges from 0-100. The lower the score, the lower the disease impact. Minimal (score<25), mild (score=25–49), moderate (score=50–74) and severe (score>74) disease impact. |
| Modified Fatigue Impact Scale (MFIS) [27] | Examines presence of fatigue across 21 items, each of which is scored 0 (no problem) to 4 (extreme problem), providing a continuous scale of 0–160. It is composed of three subscales that describe how fatigue impacts upon cognitive (10 items), physical (9 items) and psychosocial functioning (2 items). | The Total MFIS score can range from 0-84. A total score of 38 or more is indicative of fatigue. |
| Hospital Anxiety and Depression Scale (HADS) [28] | Assesses psychological distress with two sub-scales of anxiety and depression and is used widely among people with physical illnesses. | Each item is scored from 0-3 with a total score ranging from 0-21 for both anxiety and depression subscales. Higher scores indicative of greater anxiety or depression. Scores from 8-10 on each scale indicate *possible* clinical disorder (Borderline).Scores from 11-21 indicate *probable* disorder. |
| Palliative care Outcomes Scale (POS) and POS Symptoms Scale (POS-S) [29] [30] | POS records 10 items important in palliative care, including pain, symptoms, anxiety, information, depression and practical needs. POS-S records 18 symptoms relevant to palliative care. All items are assessed on a likert scale where 0=best to 4=worst. | Higher scores indicate greater levels of symptom-related distress |
